# Exposure to phthalates, potential endocrine disruptors, in an infant cohort in Modena, Italy

**DOI:** 10.1093/eurpub/ckac129.568

**Published:** 2022-10-25

**Authors:** C Lugli, L Palandri, A Ferrari, R Barbieri, V Trevisani, E Passini, L Lucaccioni, F Facchinetti, E Righi

**Affiliations:** 1 Department of Biomedical, Metabolic and Neural Sciences, University of Modena and Reggio Emilia, Modena, Italy; 2 Department of Medical and Surgical Sciences, University of Modena and Reggio Emilia, Modena, Italy

## Abstract

Phthalates are pollutants ubiquitous in the environment. Human exposure to phthalates and their endocrine disrupting effects have been widely studied. Therefore, the European Union forbids phthalates in toys, cosmetic and kitchenware manufacturing. However, phthalate metabolites can still be found in human biological matrices. The purpose of this study is to investigate phthalate exposure over time in a group of Italian healthy newborns. In a prospective cohort study, we enlisted 187 women who gave birth in the University Hospital of Modena, Italy, between January 2019 and May 2020. Urine samples from women after delivery and from their infants at birth, 3 and 6 months were collected and 8 metabolites of 6 phthalates were analysed. Descriptive statistics were calculated and preliminary correlation coefficients tests were performed. Monoethylphthalate (MEP) was always detectable in urine samples. MEP, monomethylphthalate and diethylhexylphthalate metabolites showed an increasing trend over time, while monobutylphthalate and monobenzylphthalate showed decreasing levels over time. Associations between levels of phthalates metabolites in mother and infant pairs at birth were found for a few metabolites, while metabolites in infant samples at 3 and 6 months appeared often significantly associated. Infants’ phthalate exposure in Modena is still high and prolonged over time, even to those more toxic and strictly regulated. As phthalates presence in indoor environment can be a risk factor especially for the most fragile groups of population, such as children, public Health campaigns addressing childbearing age women should stress about the risk posed by these substances and how to avoid their exposure. Moreover, regulatory actions and a stricter legislation should be considered.

**Key messages:**

• In Italy infant exposure to phthalates, including those strictly forbidden, appears still high and continuous over time.

• Public heath intervention and stricter regulatory actions should be considered.

